# Supplementary material for: Experience with Cutaneous Manifestations in COVID-19 Patients during the Pandemic
Source: J Clin Med. 2022 Jan 25;11(3):600. doi: 10.3390/jcm11030600 (PMC8836359; doi:10.3390/jcm11030600)
Supplement: Supplementary file 1 [file jcm-11-00600-s001.zip › jcm-1549359 - supplementary.pdf]

SUPLEMENTARY MATERIAL

Cuestionario COVID-piel 1ª visita

Datos ambientales

Profesión

Tu respuesta

¿Ha practicado deporte en el último mes?

- ◯ Sí
- ◯ No

En caso afirmativo, especificar cuál

Tu respuesta

¿Va descalzo habitualmente?

- ◯ Sí
- ◯ No

¿Es fumador?

- ◯ Sí
- ◯ No

En caso afirmativo, especificar cantidad (cigarrillos/día)

- ◯ <10 cig/día
- ◯ 10-20 cig/día
- ◯ >20 cig/día

¿Ha estado en contacto con alguien con sintomatología sugestiva o diagnosticado de COVID?

- ○ Sí
- ○ No

Antecedentes personales

¿Padece alguna enfermedad autoinmune/reumatológica?

- ○ Sí
- ○ No

En caso afirmativo, especifique cuál

- ☐ Lupus eritematoso sistémico
- ☐ Síndrome antifosfolípido
- ☐ Síndrome Sjögren
- ☐ Esclerodermia
- ☐ Miopatía inflamatoria (dermatomiositis, polimiositis)
- ☐ Arteritis de células gigantes/polimialgia reumática
- ☐ Artritis reumatoide
- ☐ Vasculitis
- ☐ Sarcoidosis
- ☐ Enfermedad inflamatoria intestinal (colitis ulcerosa/enfermedad de Crohn)
- ☐ Enfermedad celiaca
- ☐ Asma
- ☐ Diabetes mellitus tipo 1

¿Padece alguna enfermedad trombótica?

- ○ Sí
- ○ No

En caso afirmativo, especifique cuál

- [ ] Trombosis venosa profunda
- [ ] Tromboembolismo pulmonar
- [ ] Isquemia arterial
- [ ] Embolia sistémica
- [ ] Accidente cerebrovascular (ictus isquémico)
- [ ] Fibrilación auricular

¿Está en tratamiento anticoagulante?

- [ ] Sintrom
- [ ] Anticoagulante de acción directa
- [ ] Heparina
- [ ] No

¿Toma tratamiento antiagregante?

- [ ] AAS
- [ ] Clopidogrel
- [ ] Otros (prasugrel, ticagrelor...)
- [ ] No

¿Toma algún tratamiento inmunosupresor/inmunomodulador?

- [ ] Hidroxicloroquina
- [ ] Corticoides
- [ ] Otros inmunosupresores
- [ ] No

¿Toma antivirales para tratamiento de VIH/VHC/VHB?

- ○ Sí
- ○ No

¿Toma medicación anticonceptiva hormonal?

- ○ Sí
- ○ No

¿Toma antiinflamatorios de forma crónica (AINEs)?

- ○ Sí
- ○ No

¿Tiene alguno de los siguientes antecedente dermatológico?

- ☐ Psoriasis
- ☐ Eccema
- ☐ Urticaria
- ☐ Erupción medicamentosa
- ☐ Púrpura/vasculitis
- ☐ Fotodermatosis
- ☐ No

¿Tiene antecedentes familiares de alguna de las siguientes enfermedades?

- ☐ Enfermedades dermatológicas
- ☐ Enfermedades autoinmunes
- ☐ Enfermedades trombóticas/vasculares
- ☐ No

Clínica cutánea

Fecha de aparición

DD MM AAAA

__ / __ / ____

Localización

- [ ] Cabeza
- [ ] Tronco
- [ ] Extremidades superiores
- [ ] Extremidades inferiores
- [ ] Manos
- [ ] Pies
- [ ] Palmas y/o plantas
- [ ] Genitales
- [ ] Mucosas

Tipo de erupción

- ◯ Perniosis-like
- ◯ Vesiculosa
- ◯ Urticariforme
- ◯ Maculopapular
- ◯ Livedoide/necrótica
- ◯ Enantema
- ◯ Otro: ______________

En caso de ser maculo-papular, ¿se corresponde con alguna de las siguientes manifestaciones?

- ◯ Pitiriasis rosada-like
- ◯ Eritema multiforme-like
- ◯ Eritema elevatum diutinum-like
- ◯ Perifolicular
- ◯ Purpúrica
- ◯ Flexural
- ◯ Morbiliforme
- ◯ Fotodistribuido
- ◯ Otro: ____________

¿La clínica cutánea es sospechosa de toxicodermia?

- ◯ Sí
- ◯ No

Síntomas cutáneos

- ☐ Prurito
- ☐ Escozor
- ☐ Dolor
- ☐ Ninguno
- ☐ Otro: ____________

Clínica COVID

¿Ha tenido sintomatología sugestiva de COVID?

- ○ Sí
- ○ No

En caso afirmativo, ¿en qué fecha aparecieron los síntomas?

DD MM AAAA

__ / __ / ____

En caso afirmativo, especificar cuál

- ☐ Tos
- ☐ Disnea
- ☐ Fiebre
- ☐ Mialgias/astenia/malestar general
- ☐ Cefalea
- ☐ Náuseas/vómitos/diarrea
- ☐ Anosmia/ageusia
- ☐ Otro: ____________________

Cuestionario COVID-piel 2ª visita

Versión 1.1

Fecha de la visita

DD MM AAAA

__ / __ / ____

Nº identificación

Tu respuesta

Evolución de la clínica cutánea

¿Ha mejorado la clínica cutánea?

- ◯ Totalmente
- ◯ Parcialmente
- ◯ No ha mejorado

¿Ha recibido algún tratamiento dermatológico?

- ☐ Corticoides tópicos
- ☐ Corticoides orales
- ☐ Antihistamínicos
- ☐ No

¿Se la ha realizado biopsia cutánea?

- ◯ Sí
- ◯ No

En caso afirmativo, especificar diagnóstico histológico

Tu respuesta ____________________

Evolución de la clínica COVID

¿Ha precisado ingreso por la sintomatología COVID?

- ◯ Sí
- ◯ No

¿Ha requerido UCI?

- ◯ Sí
- ◯ No

¿Ha fallecido?

- ◯ Sí
- ◯ No

¿Ha recibido tratamiento específico para la infección COVID?

- ◯ Sí
- ◯ No

En caso afirmativo, especificar cuál

- ☐ Hidroxicloroquina
- ☐ Azitromicina
- ☐ Lopinavir/ritonavir
- ☐ Tocilizumab
- ☐ Corticoides (BOLO)
- ☐ Corticoides (NO bolo)

Diagnóstico COVID

¿Ha tenido alguna prueba positiva para COVID?

- ○ Sí
- ○ No
- ○ No se ha realizado ningún test

En caso de prueba positiva, especificar cuál

- ☐ PCR de frotis nasofaríngeo
- ☐ Test rápido serológico
- ☐ Serología en sangre IgM
- ☐ Serología en sangre IgG

¿Cuál ha sido la fecha del positivo?

DD MM AAAA

__ / __ / ____

Parámetros analíticos

Glucosa

Tu respuesta

Creatinina

Tu respuesta

Urea

Tu respuesta

GOT

Tu respuesta

GPT

Tu respuesta

GGT

Tu respuesta

LDH

Tu respuesta

PCR

Tu respuesta

Ferritina

Tu respuesta

Vitamina D

Tu respuesta

Leucocitos

Tu respuesta

Linfocitos

Tu respuesta

Hemoglobina

Tu respuesta

Plaquetas

Tu respuesta

Dímero D

Tu respuesta

Actividad de protrombina

Tu respuesta

Anticoagulante lúpico

Tu respuesta

Anticardiolipina

Tu respuesta

Antibeta2glicoproteína

Tu respuesta

Antitrombina III

Tu respuesta

Proteína C

Tu respuesta

Proteína S

Tu respuesta

Homocisteína

Tu respuesta

Parvovirus

- ☐ IgM
- ☐ IgG
- ☐ Negativo

VHH-6

- ☐ IgM
- ☐ IgG
- ☐ Negativo

Varicela (en caso de exantema vesiculoso)

- ☐ IgM
- ☐ IgG
- ☐ PCR positiva (de vesícula)
- ☐ Negativa

Figure S1. Questionnaires (initial consultation and after 4 weeks of follow-up).

Table S1. Evolution of the incidence of cutaneous lesions associated with COVID-19 over the 4 waves of the pandemic.

**COVID-19 wave**	***n***	**Positive COVID-19 test** *n (%)*	**Maculopapular** *n (%)*	**Pseudochilblain** *n (%)*	**Vesicular** *n (%)*	**Urticarial** *n (%)*	**Livedo/necrosis** *n (%)*	**Other** *n (%)*
1 (Mar-Apr 2020)	30	4 (13.3)	14 (46.7)	8 (26.7)	1 (3.3)	2 (6.7)	4 (13.3)	1 (3.3)
2 (Jul-Aug 2020)	21	12 (57.4)	8 (38.1)	3 (14.3)	5 (23.8)	2 (9.5)	1 (4.8)	2 (9.5)
3 (Oct-Nov 2020)	7	7 (100)	3 (42.9)	1 (14.3)	2 (28.6)	1 (14.3)	0	0
4 (Jan-Feb 2021)	6	4 (66.6)	0	1 (14.3)	0	2 (28.6)	0	3 (42.9)
